# Metabolomic Reprogramming Induced by Benzo[a]pyene in Skin Keratinocytes and Protective Effects of Glutathione Amino Acid Precursors

**DOI:** 10.1111/jocd.70168

**Published:** 2025-04-10

**Authors:** Xiao Cui, Tingyan Mi, Xue Xiao, Yiying Dong, Hong Zhang, Guoqiang Chen, Xuelan Gu

**Affiliations:** ^1^ Unilever Research & Development Shanghai China

**Keywords:** 3D skin model, benzo[a]pyrene, glutathione, keratinocyte, metabolome, pollutant

## Abstract

**Background:**

Pollutant particles can penetrate and accumulate in skin, leading to excessive oxidative stress, inflammation, and skin disorders. Reduced glutathione (GSH) is considered as “the master antioxidant” and major detoxification agent.

**Aims:**

To characterize the metabolomic changes of skin keratinocytes under the pollutant benzo[a]pyrene (BaP) challenge and investigate the interventional effects of glutathione amino acid precursors (GAP).

**Methods:**

Normal human epidermal keratinocytes (NHEKs) were challenged with BaP with or without GAP treatment. GSH/GSSG levels were measured by UPLC–MS/MS. Non‐targeted metabolome analysis was conducted with UPLC‐QTOF mass spectrometry. Transcriptomics analysis was performed using RNA‐seq. DNA damage biomarker γ‐H2AX was analyzed by western blot. Reconstructed pigmented skin equivalent models (pLSE) were used for evaluating phenotypical changes.

**Results:**

One micromolar BaP exposure induced widespread metabolic reprogramming in in vitro NHEKs with over‐represented differential metabolites in pathways including purine and pyrimidine nucleotide metabolism, xenobiotic metabolism, methylation, and RNA modification, etc. GAP co‐treatment improved GSH/GSSG ratio, reduced reactive BaP metabolites, and partially reversed BaP induced metabolic and transcriptomic alterations. Western blotting further confirmed that GAP treated samples showed reduced γ‐H2AX staining. In pLSE models, GAP treatment significantly ameliorated BaP induced skin darkness and hyperpigmentation.

**Conclusions:**

In summary, GAP shows in vitro protective effects against BaP by maintaining GSH homeostasis, helping metabolic detoxification, reducing DNA damage, and is effective in preventing hyperpigmentation of skin models under pollution challenge.

According to WHO, air pollution has become one of the biggest environmental risks for human health [1]. Skin is the major outer interface between the human body and the environmental exposome, which faces direct assault by pollutants. It was found that fine particulate matter can penetrate the skin barrier, accumulate in the hair follicles, and even reach the upper dermis, which challenges the skin's defense capacity [2, 3]. Increasing epidemiological studies have revealed the causal roles of pollution in the development of skin disorders including acne, pigmented spots, accelerated extrinsic aging, and compromised barrier integrity [4, 5, 6]. Paramount new scientific understandings have been accumulated over the last decade and revealed various biological impacts of pollutants on skin at molecular, cellular, and system levels, including excessive oxidative stress, protein oxidation, lipid peroxidation, DNA damage, reduction of antioxidant capacity, reduction of ATP production, mitochondrial dysfunction, autophagy dysregulation, senescence, changes in epigenetic, transcriptomic, proteomic, metabolomic profiles, and dysfunctional microbiome networks [3, 7, 8, 9, 10, 11, 12, 13, 14, 15, 16].

However, the intertwined and complicated skin biological response to pollution makes it challenging to find effective interventions. Current anti‐pollution approaches mostly rely on antioxidation and anti‐inflammation. For example, a double‐blind, placebo‐controlled clinical trial found that dietary fish oil supplementation improved inflammatory and oxidative biomarkers in response to fine particle matters [17]. Niacinamide and 12‐hydroxystearic acid are effective in mitigating benzo[a]pyrene (BaP)‐induced hyperpigmentation possibly through boosting NAD+ and anti‐inflammation [18]. Vitamin E was found to partially restore aberrant protein expression in keratinocytes caused by diesel particle extract [12]. Several studies evaluated the protective effects of plant‐derived compounds or extracts against pollutants with antioxidative and anti‐inflammatory properties [19, 20, 21, 22, 23]. A major limitation of these studies is the neglect of the harmful metabolomic changes induced by pollutants. A few studies proposed an anti‐pollution approach through counteracting the activation of the chemical sensor aryl hydrocarbon receptor (AhR) to block xenobiotic activation of pollutant chemicals [24, 25, 26]. However, the xenobiotic response is an essential native defense mechanism against toxic chemicals. Despite generating reactive oxidative species (ROS), phase II and III xenobiotic metabolism helps eliminate the toxic metabolites [27]. Boosting the skin's antioxidant defense capacity and accelerating the xenobiotic elimination of toxic metabolites at the same time may be a more promising anti‐pollution approach for skin protection.

Reduced glutathione (GSH) is considered a major detoxification agent and “master” antioxidant. GSH is able to directly neutralize ROS, recycle primary antioxidants (vitamin C and vitamin E), and conjugate with toxic metabolites to facilitate their elimination out of cells [7]. It is therefore highly desirable to increase skin endogenous GSH to fight against pollution. In previous studies, we designed a novel blend of glutathione amino acid precursors (GAP) consisting of L‐cystine, L‐glutamine, and glycine. We proved that GAP increases cellular GSH levels and protects skin against oxidative and environmental stresses such as UV, blue light, and urban dust [7, 28]. In this study, we aimed to investigate the impact of pollutant chemicals on skin metabolome and phenotype and further evaluate the protective effects of GAP. BaP, which accounts for 27%–67% of the toxicity of air pollutants, was selected as a model pollutant chemical to challenge the in vitro skin models [25].

## 1 Materials and Methods

### 1.1 Chemicals and Reagents

Primary human epidermal keratinocytes (NHEKs) were obtained from Promocell (Heidelberg, Germany). Reconstructed human pigmented living skin equivalent models (pLSE) were provided by BioCell (MelaKuitis, Guangdong, China). NHEKs culture medium was prepared by adding 1% HKGS (Gibco, MA, US) into Epilife (Gibco, MA, US) keratinocytes culture medium with 60 μM calcium. BaP was obtained from Sigma (MO, US). GAP was prepared with L‐glutamine (Sigma, MO, US), L‐cystine (Sigma, MO, US), and glycine (Sigma, MO, US) in the weight ratio of 0.5:1:1. Fifty and 200 μM GAP (based on cystine level) were used in the experiment based on an in‐house experiment on minimal effective dose and epidermis delivery data of L‐cystine with a practical skincare formulation.

### 1.2 Keratinocyte Culture, Treatment, and Measurement

GSH/GSSG levels were measured by UPLC–MS/MS. NHEKs were seeded in a 12‐well plate with a cell density of 2 × 10^5^. On the following day, cells were treated with BaP at 10 μM in the presence or absence of 200 μM GAP in cell culture medium. After 24 h, the cells were washed with PBS twice and were then harvested with CelLytic M lysis buffer (Sigma, MO, US) using a cell scraper. The samples were kept frozen at −80°C before metabolome analysis. GSH and GSSG of cell lysates were quantified using UPLC‐MS/MS (QTRAP 6500 MS, SCIEX, USA) according to Cui et al. [7]. Three replicates were used in each group. Statistical significance was analyzed with ANOVA followed by Bonferroni post hoc test.

γ‐H2AX was analyzed by western blot. NHEKs were cultured as previously described. Then, 10 μM BaP with or without GAP was added to the culture medium. After incubation, the NHEKs were washed with PBS and lysed by 200 μL RIPA lysis buffer (containing 1× proteinase inhibitors cocktail, Thermo Scientific, MA USA). Western blot was performed to detect γ‐H2AX level in the cell lysate with Phospho‐Histone H2A.X antibody (Cell Signaling, MA, USA).

### 1.3 Non‐Targeted Metabolomic Analysis

For the metabolome analysis, a pilot study was first conducted with NHEKs treated with BaP at 1 and 10 μM. One micromolar BaP was selected for the formal experiment, which was based on OPLS‐DA results (Table S1). In the formal experiment, NHEKs treated with BaP at 1 μM in the presence or absence of 50 μM GAP had four replicates in each group. The metabolites were extracted using MeOH/ACN/H_2_O (2:2:1, vol/vol). The extracts were reconstituted in ACN/H_2_O (1:1, vol/vol). Non‐targeted metabolomic profiling was conducted using ultra performance liquid chromatography (UPLC 1290 series, Agilent, USA) coupled with quadruple time‐of‐flight (QTOF) mass spectrometry (6550 iFunnel, Agilent, USA). A Waters ACQUITY UPLC BEH amide column [Particle size, 1.7 μM; 100 mm (length) × 2.1 mm (i.d.)] was used for the LC separation. The mobile phase A was 25 mMCH_3_COONH_4_ and 25 mM NH_4_OH in water, and phase B was 100% ACN. Quality control (QC) samples were prepared by pooling aliquots of all cell samples that were representative of the cell samples. The samples were randomly injected for the UPLC‐QTOF/MS analysis. Signals in both positive ion mode (ESI+) and negative ion mode (ESI−) were acquired. Tandem mass spectrometry (MS/MS) data acquisition was performed using another QTOF mass spectrometer (Triple TOF 6600, AB SCIEX, USA). Raw data acquired from UPLC‐QTOF were converted into mzXML format using ProteoWizard. The metabolites matched to in‐house standard MS/MS library were defined as level‐1 annotated metabolites. To characterize vast unknown m/z features, the Mummichog software was employed [29]. The m/z features annotated with the Mummichog tool were defined as level‐2 putatively annotated metabolites. Level 1 and level 2 metabolites were pooled, and redundant annotations were removed. Classification of metabolites was based on annotation from HMDB (Human Metabolome Database) [30]. Data quality was assessed using principal component analysis (PCA) and QC relative standard deviation (RSD). Differential metabolites were identified using a t‐test with a cutoff of adjusted *p*‐value < 0.05. MetaboAnalyst was used for enrichment analysis with default settings [31].

### 1.4 Transcriptomics Study Using RNA‐Seq

Three replicates were used for each group of treatment. Total RNA was isolated using the Trizol (Thermo Fisher Scientific, USA) method and purified with the RNeasy MinElute Cleanup Kit (QIAGEN, Netherlands). RNA concentration is measured by NanoDrop (Thermo Fisher Scientific, USA). RNA quality is evaluated by a Agilent 2100 Bioanalyzer (Agilent Technology, USA). RNA sequence libraries were generated with standard TRUE‐seq mRNA protocols from Illumina and sequenced by Hiseq X machine (Illumina, USA) with 150 paired‐end reads. The clean reads were mapped to the human genome (version hg19) and transcriptome using the software [32]. edgeR was used for DEGs identification with the cut‐offs of |Fold change| > 1.5, FDR < 0.05 [33]. Gene Ontology (GO) enrichment analysis was performed using DAVID (Database for Annotation, Visualization, and Integrated Discovery) [34]. Gene overlap testing and visualization were conducted using the GeneOverlap package [35].

### 1.5 pLSE Model Treatment and Asessment

pLSE models were constructed in a 24‐well cell culture plate with pLSE medium (Biocell, China). After 5 days of culture since air‐lifting, 60 μM GAP blend was applied in culture medium with daily refreshment. After 3 days of treatment, 10 μL BaP at a dose of 10 μM was applied topically on the surface of the pLSE model every other day. The pLSE models were collected after another 4 days of culture. Three replicates were used for each group.

Upon harvest, skin lightness (L*) was measured using a spectrophotometer, DermalLab C4000 (Cortex Tech., Hadsund, Denmark). The L* value of each model was recorded three times from the readings of the Chroma meter. The photos of the pLSE were captured by an EOS 700D digital camera (Canon, Tokyo, Japan).

The pLSE models were immediately fixed in cold 4% neutral buffered formalin solution (Sigma Aldrich), dehydrated, and embedded in paraffin. Then, the tissues were sliced into 5 μm vertical sections and stained with Fontana‐Masson (FM) histochemical staining according to the manufacturer's instructions (Abcam, Cambridge, UK) for melanin distribution. The slides were microscopically examined, and images were captured (Leica, DM2500). The melanin content was quantified by melanin distribution tissue section staining with Image‐Pro Plus 6.0 (Media Cybernetics, MD, USA).

## 2 Results

### 2.1 Impact of BaP and GAP Treatment on GSH Homeostasis

**FIGURE 1:**
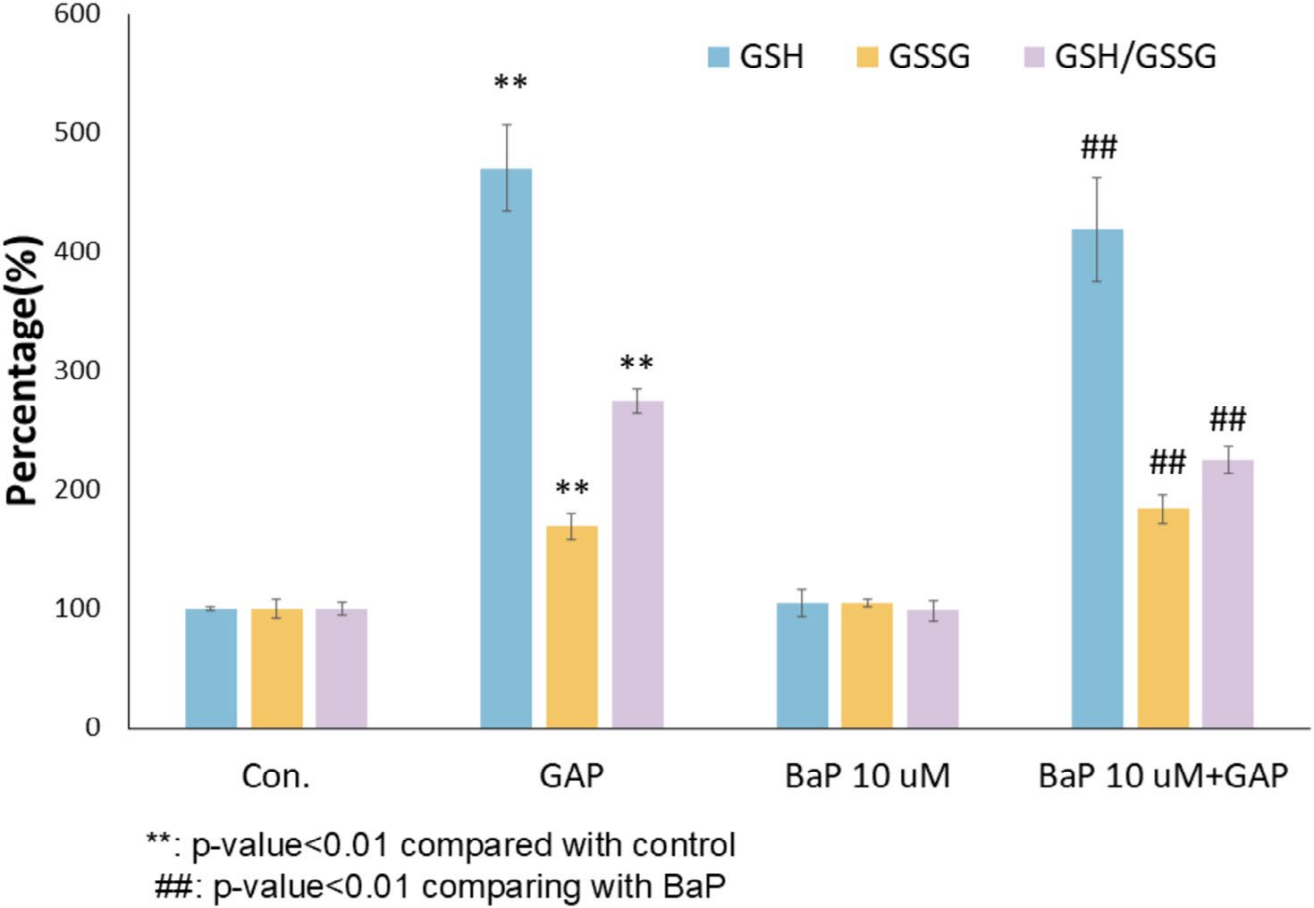
Effects of BaP and GAP on GSH redox state. GSH, GSSG, and GSH/GSSG ratio in NHEKs supplemented with 200 μM GAP with and without 10 μM BaP challenge. GSH and GSSG were quantified using UPLC‐MS/MS. GSH, GSSG, and their ratio as percentages of control. Data are represented as means ± SEM (*n* = 3); Statistical significance was analyzed with ANOVA followed by Bonferroni post hoc test.

BaP was found to have a level from 0.05 to 2.3 ng/m^3^ in ambient air. It was estimated that the 24‐h personal PAH exposure concentration was 2100 ± 1300 ng/m^3^ (1.6 part per billion) [36]. The BaP concentrations (1–10 μM BaP, ppm level) in this study were higher than real‐life pollution exposure levels but were often employed in in vitro tests in the literature as exaggerated challenge models [37, 38]. To understand the cellular glutathione redox state of NHEKs under 10 μM BaP exposure and with 200 μM GAP treatment, both reduced glutathione (GSH) and oxidized form (GSSG) were measured in the extracts of cells using UPLC‐MS/MS. The 24‐h post treatment was selected as the sampling time point to avoid the complication of the treatment effects of GAP through boosting de novo GSH synthesis and the acute activation of antioxidant defense activation through GSH regeneration [39, 40]. In the absence of BaP challenge, GAP treatment was able to increase GSH (by 370%), GSSG (by 70%), and GSH:GSSG ratio (by 175%) (Figure 1, *p* < 0.01). Under 10 μM BaP challenge, the treatment of GAP was also able to significantly increase GSH (by 300%), GSSG levels (by 75%), and GSH:GSSG ratio (by 129%) (Figure 1, *p* < 0.01). These results suggested that the GSH boosting effects of GAP are maintained in the presence of BaP challenge.

### 2.2 Non‐Targeted Metabolomics Profiling Captures Endogenous Compounds and BaP Metabolites

**FIGURE 2:**
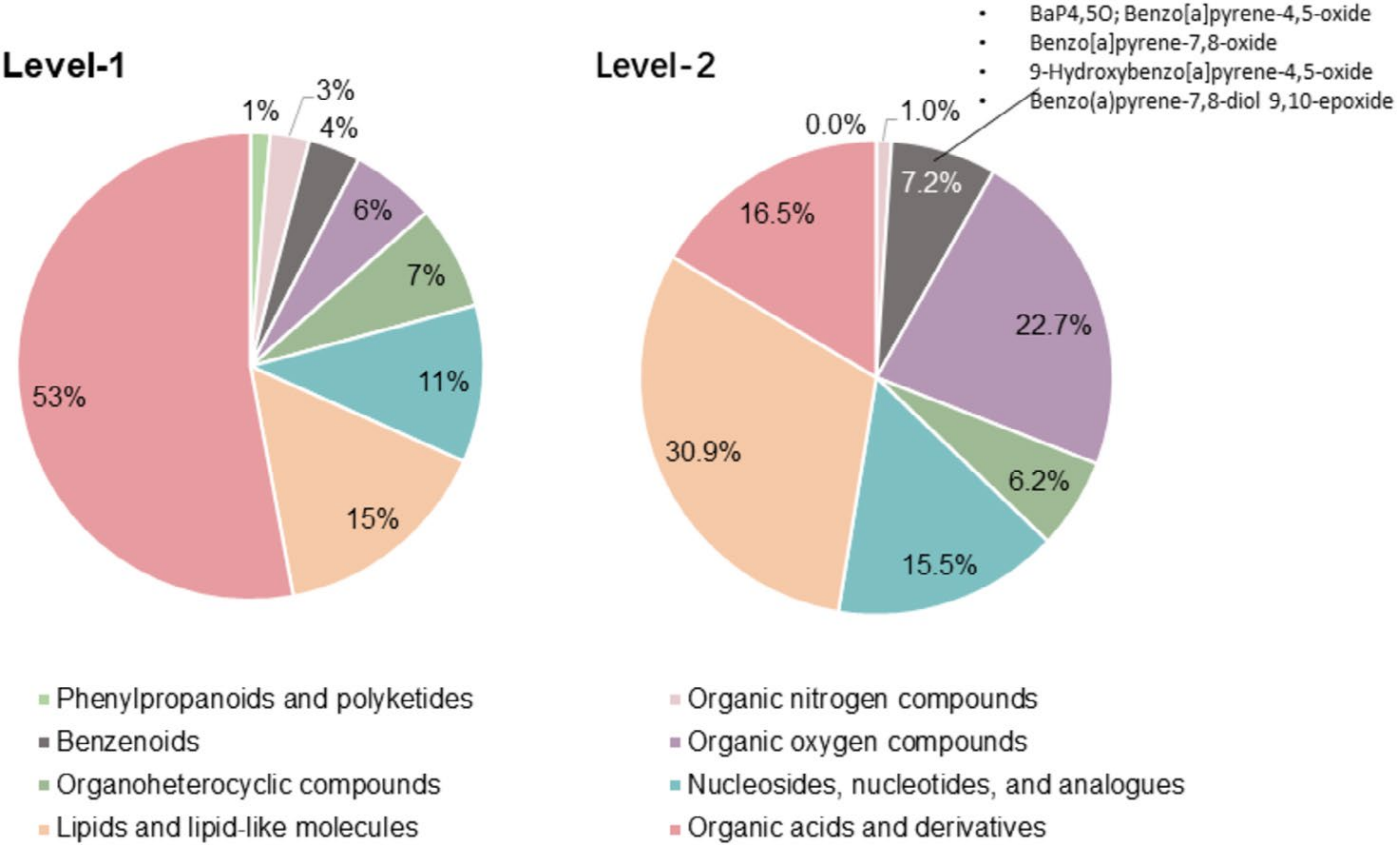
Chemical classifications of level‐1 and level‐2 annotated metabolites using ClassyFire super class. Area represents relative proportion of the metabolites of each super class. Level‐1 metabolites: Annotated with MS^2^ data; Level‐2 metabolites: Annotated with the mummichog tool.

To better characterize metabolic response to BaP and explore potential protective effects of GAP, we conducted non‐targeted metabolomic profiling with NHEKs under a lower dose BaP exposure at 1 μM BaP with or without 50 μM GAP co‐treatment. A mono‐layer NHEK model was used instead of 3D skin models with different cell types to avoid the complications of cell composition changes confounding metabolomic changes. Also, NHEKs show lower intrinsic variations between replicates than 3D skin samples. The QC metrics suggested good consistency in technical replicates. 95.6% of the m/z features from the positive model and 92.3% of the features from the negative ion model showed RSD < 30% (Figure S1). In total, we identified 463 metabolites. Three hundred thirty one metabolites were identified using MS^2^ (defined as level‐1 metabolites), and 132 metabolites were putatively annotated using mummichog (defined as level‐2 metabolites). Figure 2 shows the chemical classifications of the level‐1 and level‐2 metabolites. Four BaP metabolites were identified as level‐2 metabolites, including benzo[a]pyrene‐4,5‐oxide, benzo[a]pyrene‐7,8‐oxide, 9‐hydroxybenzo[a]pyrene‐4,5‐oxide, and benzo(a)pyrene‐7,8‐diol 9,10‐epoxide.

### 2.3 BaP Exposure Reprogram Metabolism of NHEKs

**FIGURE 3:**
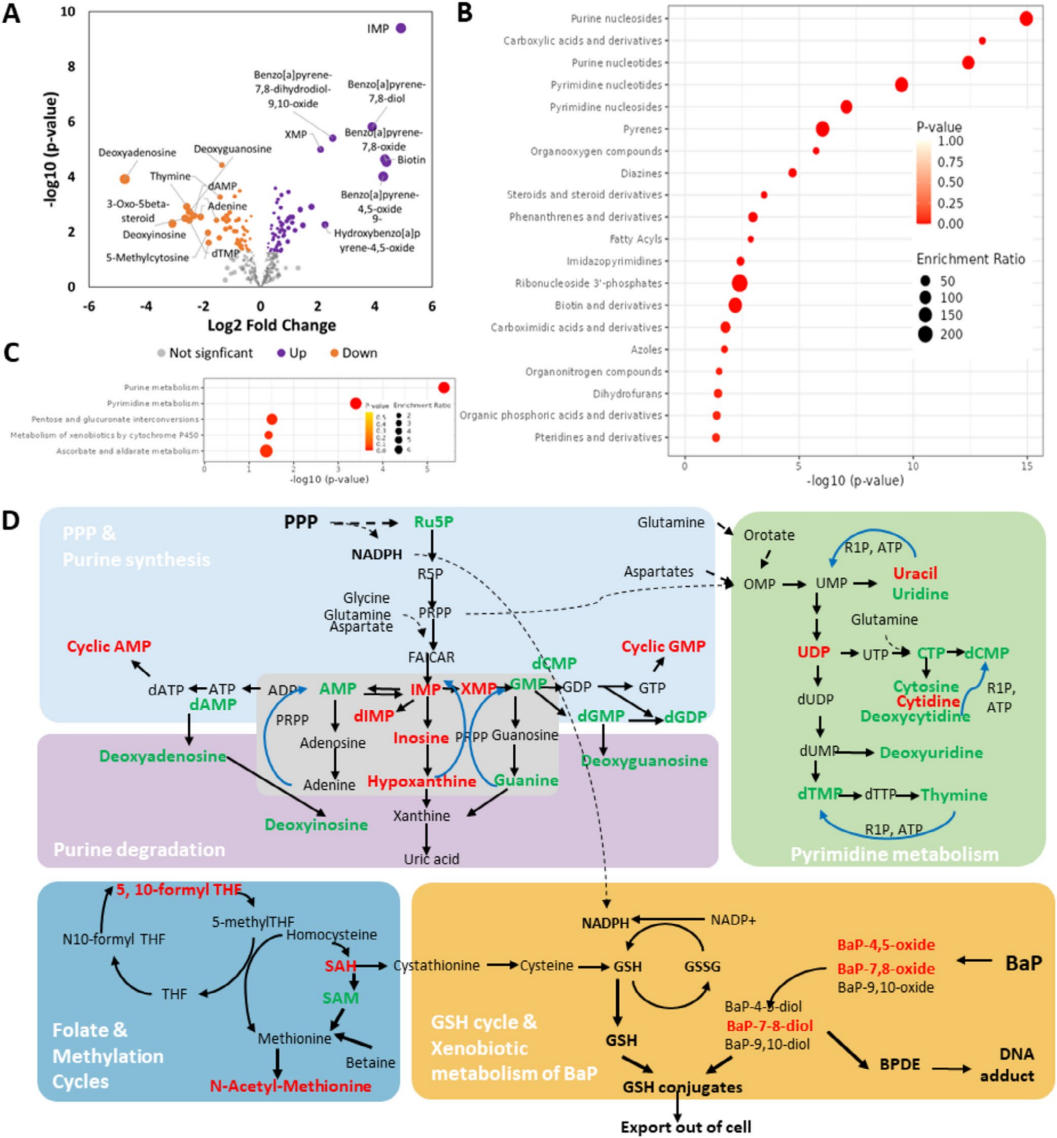
BaP exposure reprograms metabolism of keratinocytes. (A) Volcano plot of metabolites significantly altered by BaP exposure (*p*‐value < 0.05, *n* = 4, *t*‐test, Table S1); (B) Significant over‐represented chemical classes of BaP altered metabolites (*p*‐value < 0.05, global‐test, Table S2); (C) Significantly enriched pathways of BaP altered metabolites (*p*‐value < 0.05, global‐test, Table S3); (D) BaP exposure reprograms pentose phosphate and nucleotide metabolism. ADP, adenosine diphosphate; AMP, adenosine monophosphate; ATP, adenosine triphosphate; BPDE, Benzo (a)pyren‐7,8‐dihydrodiol‐9,10‐epoxide; CTP, cytidine triphosphate; dADP, deoxyadenosine diphosphate; dAMP, deoxyadenosine monophosphate; dCMP, deoxycytidine monophosphate; dGDP, deoxyguanosine diphosphate; dGMP, deoxyguanosine monophosphate; dTMP, deoxythymidine monophosphate; dUDP, deoxyuridine diphosphate; FAICAR, 5‐formamidoimidazole‐4‐carboxamide ribotide; GMP, guanosine monophosphate; GPX, glutathione peroxidase; GR, glutathione reductase; IMP, inosine monophosphate; PRPP, phosphoribosyl pyrophosphate; R1P, ribose‐1‐phosphate; R5P, ribose‐5‐phosphate; Ru5P, ribulose‐5‐phosphate; SAH, s‐adenosyl homocysteine; SAM, s‐adenosyl methionine; THF, tetrahydrofolate; UDP, uridine diphosphate; UMP, uridine monophosphate; XMP, xanthosine monophosphate.

In total, 110 of the 463 identified metabolites were significantly altered in NHEKs under BaP exposure, including 61 up‐regulated and 49 down‐regulated metabolites (Figure 3A, Table S1). The top 10 significantly altered metabolites were highlighted in the volcano plot, including 8 upregulated ones (IMP；Benzo[a]pyrene‐7,8‐diol； Benzo[a]pyrene‐7,8‐dihydrodiol‐9,10‐oxide；XMP；Biotin；Benzo[a]pyrene‐7,8‐oxide；Benzo[a]pyrene‐4,5‐oxide; 3‐Phospho‐D‐glycerate) and 2 downregulated metabolites (LysoPC(14:0)；Deoxyadenosine). Enrichment analysis showed that the differential metabolites altered by BaP were overrepresented with chemical classes including purine nucleotides, carboxylic acids and derivatives, pyrimidine nucleotides/nucleosides, pyrenes, diazines, steroids, and steroids derivatives, and forth. (Figure 3B, Table S2). Further pathway analysis revealed that BaP significantly altered 5 metabolic pathways, such as purine metabolism, pyrimidine metabolism, pentose glucoronate interconversion, and metabolism of xenobiotics by cytochrome P450 (Figure 3A, Table S3).

The interconnected pathways affected by BaP were summarized in Figure 3D. It was found that the non‐cyclic deoxynucleotides/deoxynucleosides were all reduced by BaP treatment. Cyclic AMP (cAMP) and cyclic GMP (cGMP) were also significantly increased by BaP. On the other hand, several intermediate metabolites in purine biosynthesis and degradation were reduced, such as inosine monophosphate (IMP), dIMP, inosine, hypoxanthine, and xanthosine monophosphate (XMP). In the methylation cycle, SAH (s‐adenosyl homocysteine) was upregulated by BaP, while SAM (s‐adenosyl methionine) was downregulated. Interestingly, N6‐methyladenosine (m6A) and isopentenyladenosine (i6A) were increased upon BaP treatment, which are related to RNA modification (Figure S2).

### 2.4 GAP Partially Restores Metabolomic Changes Induced by BaP, Reduces Toxic BaP Metabolites, and DNA Damage Markers in NHEK

**FIGURE 4:**
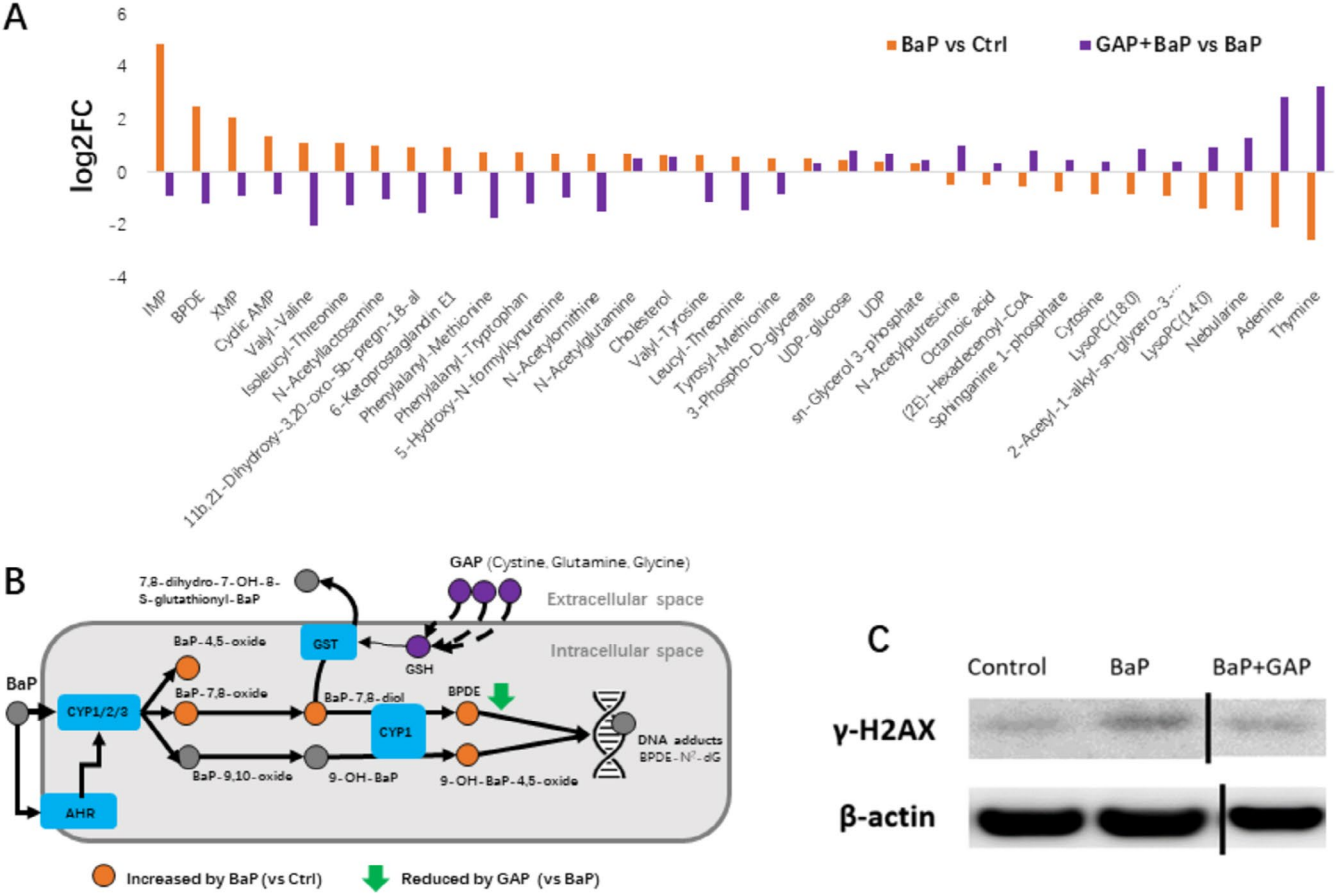
GAP treatment partially restored metabolic alterations under BaP exposure. (A) Fold changes of overlapping significant differential metabolites of BaP versus Ctrl and BaP + GAP co‐treatment versus BaP (*p* < 0.05, *n* = 4, *t*‐test); (B) GAP facilitates the xenobiotic detoxification process of BaP and reduces BPDE; (C) Western Blotting of the DNA damage marker γ‐H2AX.

In the next, we explored whether GAP treatment is effective to mitigate BaP induced metabolic changes. In total, 74 metabolites were significantly altered by GAP + BaP co‐treatment compared to BaP exposure alone (Table S4). Thirty three overlapping differential metabolites were found in GAP + BaP differential metabolites (vs. BaP) and BaP differential metabolites (vs. Ctrl) (Figure 4A, Table S5). Twenty seven metabolites (81.8%) showed reversed direction of changes, while 7 metabolites (18.2%) showed the same directions of changes. It is noticeable that top changed metabolites altered by BaP were partially restored by GAP, including IMP, XMP, benzo[a]pyrene‐7,8‐dihydrodiol‐9,10‐oxide (BPDE), and LysoPC(14:0) (Figures 3A and 4A). It is noteworthy that BPDE is the most toxic metabolite of BaP, which can bind to DNA, form DNA adducts, and cause mutations [28, 29]. It was found to be increased more than 5.7 times by BaP treatment and was reduced by GAP by 56.5% (natural scale of peak areas). Several dipeptides were found to be significantly increased by BaP but were reduced by GAP treatment, including tyrosyl‐methionine, leucyl‐threonine, valyl‐tyrosine, isoleucyl‐threonine, and valyl‐valine. GAP also restored the decline of glycerophospholipids (LysoPC(18:0), LysoPC(14:0)) and sphingosine 1‐phosphate. To further validate whether the reduction of BPDE is helpful to prevent DNA damage, we conducted western blot of γ‐H2AX, which is a sensitive molecular marker of DNA damage and repair. It was found that BaP treatment induced expression of γ‐H2AX, which was abrogated by GAP treatment (Figure 4C). Taken together, these results indicate that GAP partially restored major metabolic changes of BaP exposure, facilitated xenobiotic elimination of BaP metabolites, and helped to mitigate DNA damage.

### 2.5 GAP Partially Restores Transcriptomic Changes in NHEKs

Given the metabolic restoration effects of GAP against BaP exposure, we further conducted a transcriptomic study using RNA‐seq to explore if GAP could reverse BaP‐induced gene expression changes. In total, we found 32 overlapping differentially expressed genes (DEGs) affected by both GAP and BaP treatment, with 28 of them showing a reversed direction of changes (Figure S3A). Gene set overlap analysis revealed that this reversed overlap pattern of up‐ and down‐regulated genes by GAP and BaP treatment was significant (Figure S3B). GO enrichment analysis showed that GAP‐reversed genes are mainly involved in keratinization, epidermis development, cell fate determination, and so forth. (Figure S3C). Given the limited DEGs identified, further multi‐omics analysis was not conducted.

### 2.6 GAP Ameliorates BaP‐Induced Hyperpigmentation in 3D Reconstructed Skin Models

**FIGURE 5:**
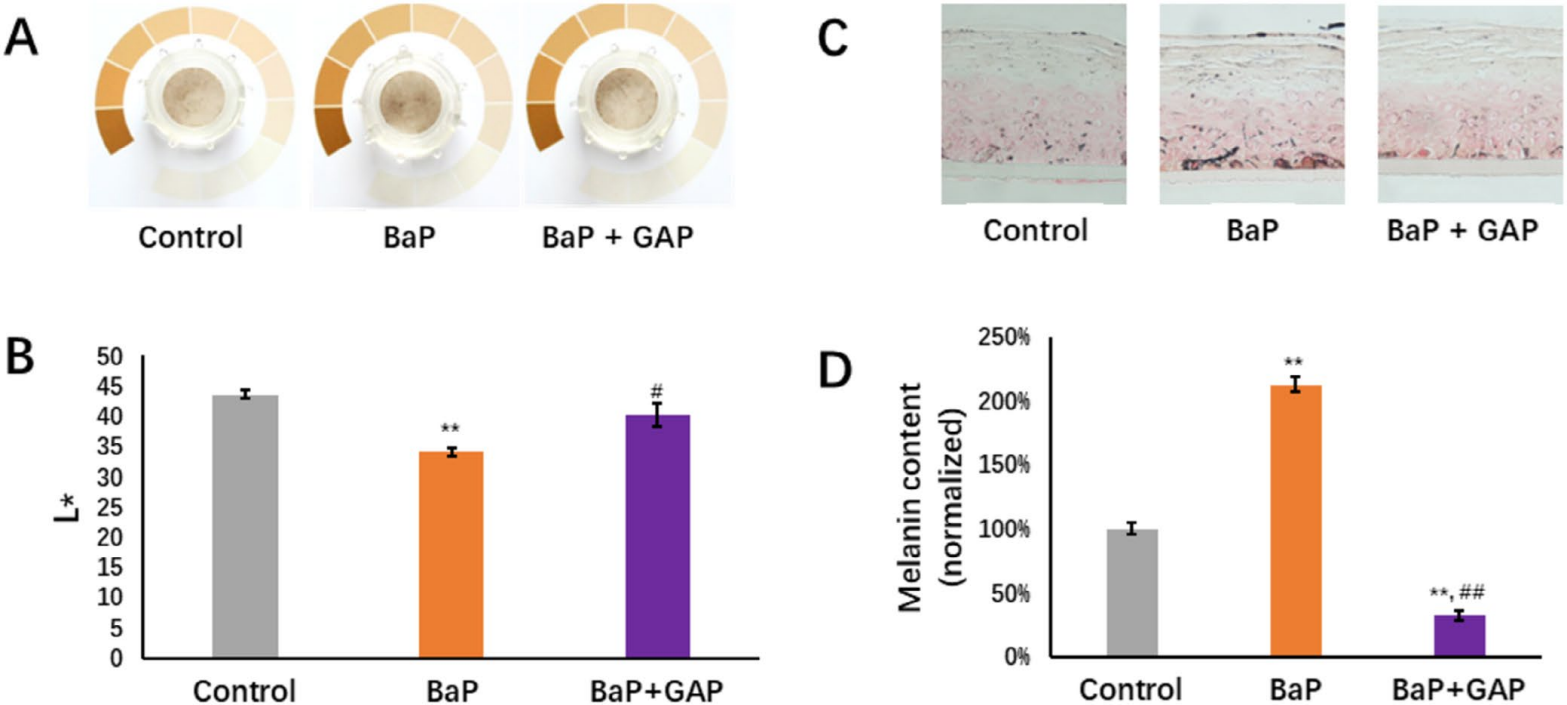
Effects of BaP and GAP on appearance and pigmentation of pLSE model. (A) Representative top view of photos of pLSE models. (B) L* values (skin brightness) measured by spectrophotometer. (C) Melanin deposition (FM staining); (D) Melanin content (normalized to control group). Data are represented as means ± SEM (*n* = 3), Statistical analysis was performed using one‐way ANOVA followed by Bonferroni post hoc test. *p‐value < 0.05; **p‐value < 0.01 compared with control; ^#^ *p*‐value < 0.05 comparing with BaP; ^##^ *p*‐value < 0.01 comparing with BaP.

It is reported that pollutant exposure causes skin hyper‐pigmentation through AhR activation; our previous study indicated that BaP evidently induced the darkening level in a pLSE model [26]. To explore the impact of BaP and GAP treatment on the phenotypical end‐point of the skin model, we further evaluated GAP's potential skin benefit by measuring the changes in skin surface appearance and melanin distribution using the pLSE model. It was found that pLSE models with the presence of 10 μM BaP topically applied were darker than the control (Figure 5A). The lightness of pLSE models was further quantified using a spectrophotometer. BaP caused a significant decrease in L* value (△L*: −9.6, *p*‐value < 0.01, compared to control group), while GAP treatment resulted in a significant increase in L* value (△L*: 6.1, *p* < 0.05, compared to BaP group) (Figure 5B). Fontana‐Masson (FM) staining further showed increased melanin deposition in pLSE models challenged by BaP; in particular, in the basal layer, while fewer melanin deposits were observed in the control and GAP‐treated models (Figure 5C). Quantification of melanin staining confirmed that BaP leads to a significantly higher level of total melanin than the control group (by 112%, *p* < 0.01) and the GAP‐treated group (by 85%, *p* < 0.01) (Figure 5D).

## 3 Discussion

Various studies showed that pollutant chemicals trigger excessive oxidative stress, leading to cellular damage and inflammation ramifications in skin cells. However, the impact of pollutants on normal human skin cell metabolism is less characterized. In this study, we found that BaP exposure at 10 μM did not significantly change the redox state (GSH:GSSG ratio) of the NHEKs, while an even lower exposure level of BaP at 1 μM was able to induce extensive disturbances to the metabolome of NHEKs. The sensitive metabolic response of NHEKs to lowdoses of BaP exposure may be explained by high metabolic activities in keratinocytes, which are needed for the turnover of the epidermis, wound healing, and xenobiotic metabolism [40, 41, 42]. A sensitive response to low‐dose BaP exposure was found in liver cells, which is attributed to the high expression of cytochrome P450 (*CYP*) genes initiating the xenobiotic metabolism [43]. Extrahepatic expression of CYP genes was found in the epidermis with six CYP genes upregulated during keratinocyte differentiation [44]. Taken together, these results imply that the metabolic activities in keratinocytes are sensitive to pollutant chemical exposure. It is reported that skin cells can regenerate GSH by NADPH through the pentose phosphate pathway within minutes of UV exposure. Further time course studies are needed to characterize the dynamic changes of GSH:GSSG in NHEKs under BaP challenges.

Using non‐targeted metabolomic profiling, our study provided a global snapshot of keratinocyte metabolic changes under BaP exposure. In total, 110 identified metabolites significantly altered by 1 μM BaP exposure in NHEKs are involved in purine/pyrimidine metabolism, xenobiotic processes, the pentose phosphate pathway, and forth. Our study showed that the deoxynucleotides/deoxynucleosides were all reduced by BaP. This might be due to the DNA damage and cell cycle arrest caused by BaP. The cellular pool of deoxyribonucleotide is tightly controlled to ensure efficient and precise genome duplication during the S‐phase [45]. On the other hand, inosine monophosphate (IMP), dIMP, inosine, hypoxanthine, and xanthosine monophosphate (XMP) were all significantly increased. IMP is an intermediate metabolite in purine salvage and degradation. The increase of IMP and its metabolites may indicate increased DNA deamination under BaP stress, which occurs spontaneously in cells and is enhanced by exposure to pollution [46]. Changes in purine and pyrimidine metabolism were also observed in the BaP‐exposed lung cancer cell line (H460) and the mouse skin model of carcinogenesis [47, 48]. Taken together, we observed extensive metabolic rewiring reflecting the DNA damage response caused by BaP exposure. Air pollution exposure is reported to affect the epigenome and epi‐transcriptome [transcriptome [transcriptome [transcriptome [49, 50]. In the methylation cycle, S‐adenosylmethionine (SAM) is a major methyl donor, while S‐adenosylhomocysteine (SAH) is an inhibitor of transmethylations, and the ratio SAM/SAH is regarded as an index of methylating capacity [51, 52]. Our observation of reduced SAM and increased SAH implies that BaP may reduce the methylation capacity of NHEKs. N6‐methyladenosine (m6A) and isopentenyladenosine (i6A) were found increased upon BaP exposure in our study. m6A is the most prevalent methylated nucleoside in eukaryotic mRNA that plays important roles in the rapid reshaping of the transcriptome to adopt stress challenges [53, 54]. The i6A modified tRNA is required for the synthesis of selenoproteins and mitochondrial proteins, which are required for maintaining redox and metabolic homeostasis [55, 56]. The increased m6A and i6A in the metabolome may come from the degradation of modified RNAs. Further studies are needed to understand the context‐specific changes of RNA/DNA modification under BaP exposure and the crosstalk between the metabolome and the epigenome/epi‐transcriptome.

Our findings also implies that the primary antioxidant (e.g., ascorbic acid) which do not have detoxification function may be not effective to protect the skin cell against pollutant chemicals, given that low‐dose BaP exposure caused extensive metabolic shift without altering redox status. Another limitation of ascorbic acid is that its oxidized product is a source of oxoaldehyde and glycative stress [57]. Reduced glutathione (GSH) could conjugate with BaP‐oxide and facilitate its elimination, making it an appealing agent for skin protection against pollutants. Previously, we proved that GAP increases the cellular GSH level and protects skin against oxidative stress and UV [7, 28]. This metabolomic analysis further provides proof‐of‐concept evidence supporting protective effects of GAP against pollutant BaP through metabolic restoration. Our results showed that GAP treatment partially restored the metabolic changes induced by BaP, including BPDE, IMP, and XMP. This implied less DNA damage due to better eliminating toxic BaP metabolites. Indeed, we found that GAP treatment reduced staining of DNA damage biomarker γ‐H2AX. Previous study showed that pollutant particles induce pigmentation through AhR signaling and α‐MSH paracrine levels in keratinocytes [26]. Using RNA‐seq, we further find that GAP treatment could partially restore gene expression changes induced by BaP. However, since low dose was used in this study, the limited number of DEGs were not useful for multi‐omics analysis. Future studies are needed to investigate the associations of metabolomic changes and gene expression of BaP and GAP treatment at higher doses.

Using a 3D reconstructed skin model, we found that GAP is effective in protecting skin against BaP‐induced hyper‐pigmentation. Several mechanisms may explain the protective effects of GAP in defending skin tone. First, GAP boosts endogenous GSH, which not only reduces oxidative stress but also regulates the melanin synthesis pathway [58]. Second, GAP can accelerate GSH‐dependent detoxification pathway and reduce the toxic metabolites of pollutants [59, 60]. This helps to prevent DNA damage and reduce AHR signaling that triggers hyper‐pigmentation.

## 4 Conclusions

In this study, we found that low‐dose BaP exposure was able to reprogram the metabolome of NHEKs without changing the redox status. We identified 110 metabolites significantly changed by BaP, which are involved in purine/pyrimidine nucleotide metabolism, xenobiotic metabolic pathways, methylation, and RNA modification. The study revealed protective effects of GAP against BaP through metabolic restorations, reducing DNA damage, and supporting the GSH booster as a novel anti‐pollution approach through metabolic detoxification. The proof‐of‐concept study using a 3D reconstructed skin model supported the efficacy of GAP in defending skin tone against BaP‐induced hyperpigmentation. Future studies are needed to evaluate the efficacy of topical GAP formulation against pollutant exposure with more real‐life relevant skin models.

## Author Contributions

Xiao Cui and Tingyan Mi: conceptualization. Xiao Cui, Tingyan Mi, Yiying Dong and Guoqiang Chen: methodology, validation, formal analysis and investigation. Xuelan Gu: resources, supervision, project administrationa and writing – review and editing. Xiao Cui: software, data curation and visualization. Xiao Cui, Xiao Xue and Hong Zhang: writing – original draft preparation. Tingyan Mi: funding acquisition. All authors have read and agreed to the published version of the manuscript.

## Conflicts of Interest

The authors declare no conflicts of interest.

## Supporting information


**Figure S1** QC of metabolomics data.

**Figure S2** Changes of m6A, i6A upon BaP exposure.

**Figure S3** RNA‐seq results of BaP treatment and GAP intervention using NHEKs model.

**Table S1** Summary of OPLS‐DA model statistics.
**Table S2** Differential metabolites upon BaP treatment (*p* < 0.05).
**Table S3** Chemical class enrichment results of differential metabolites upon BaP treatment.
**Table S4** Pathway enrichment results of differential metabolites upon BaP treatment.
**Table S5** Differential metabolites of GAP intervention (vs BaP, *p* < 0.05).
**Table S6** Overlapping differential metabolites of BaP treatment and GAP intervention.

## Data Availability

The data that support the findings of this study are available from the corresponding author upon reasonable request.
