# Supplementary figures and images for: Metabolomic Reprogramming Induced by Benzo[a]pyene in Skin Keratinocytes and Protective Effects of Glutathione Amino Acid Precursors
Source: J Cosmet Dermatol. 2025 Apr 10;24(4):e70168. doi: 10.1111/jocd.70168 (PMC11984496; doi:10.1111/jocd.70168)

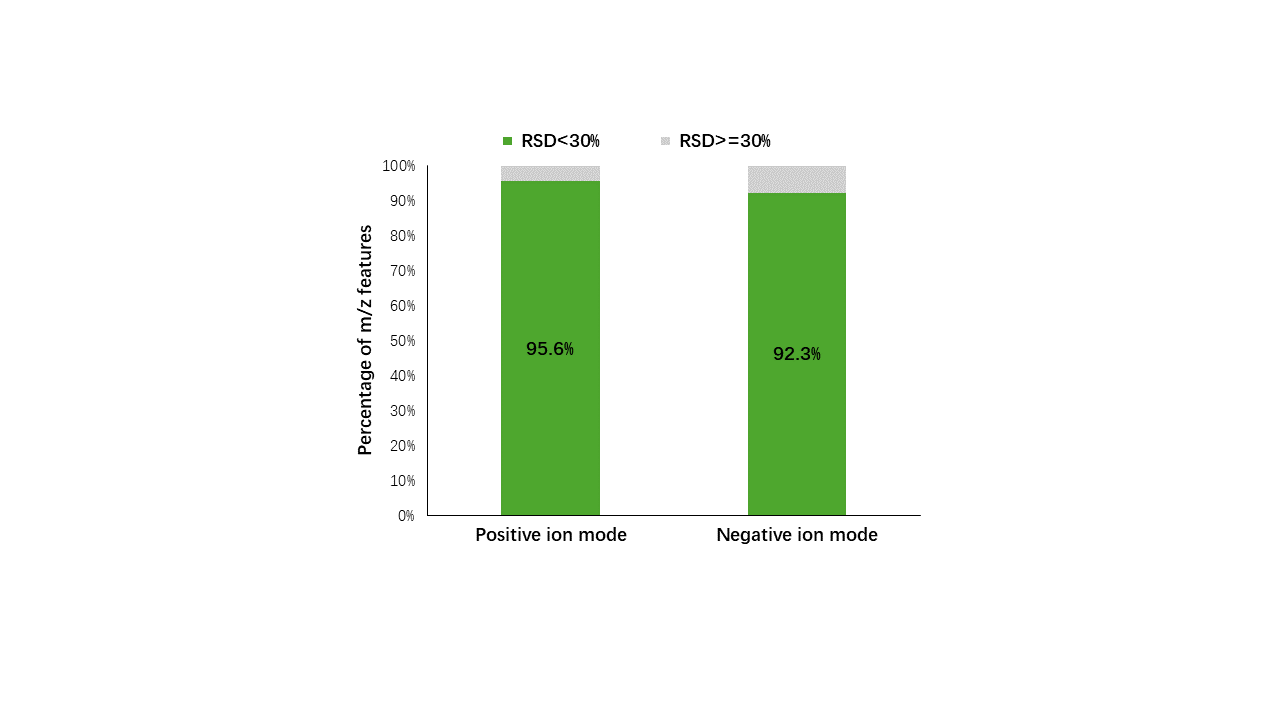

Supplement: Supplementary file 1 — Figure S1 QC of metabolomics data. [file JOCD-24-e70168-s004.png]

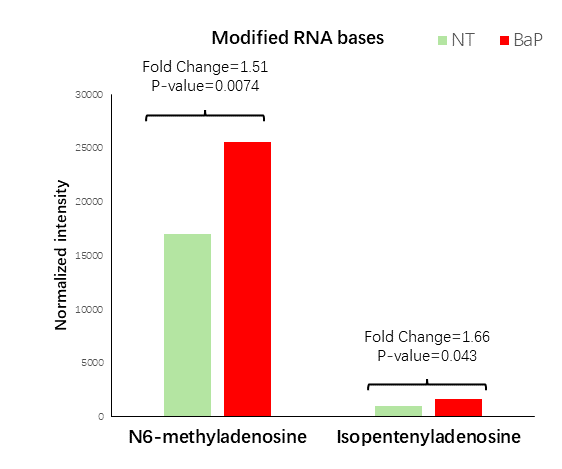

Supplement: Supplementary file 2 — Figure S2 Changes of m6A, i6A upon BaP exposure. [file JOCD-24-e70168-s001.png]

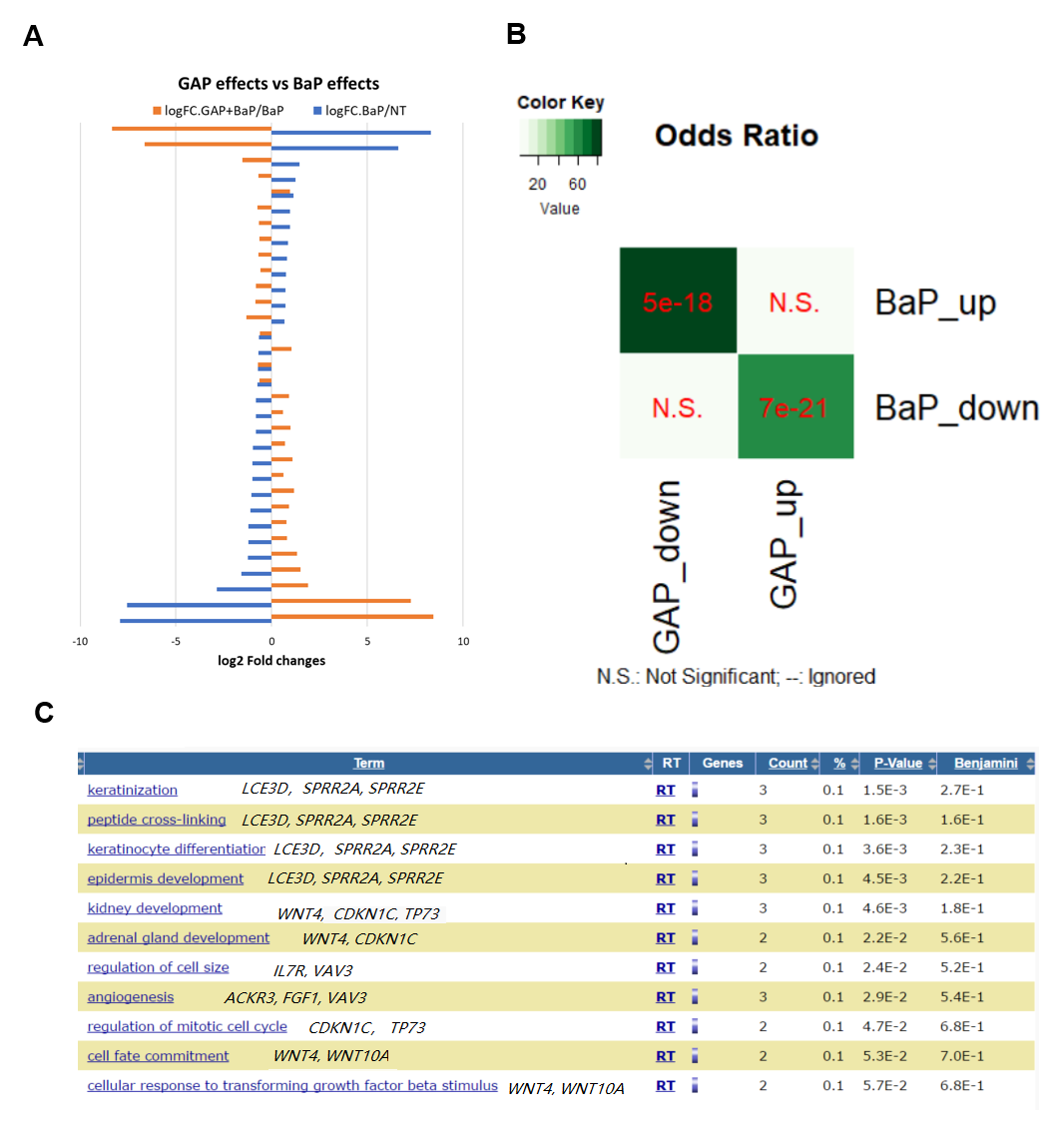

Supplement: Supplementary file 3 — Figure S3 RNA‐seq results of BaP treatment and GAP intervention using NHEKs model. [file JOCD-24-e70168-s002.png]
